# Obesity and Modifiable Cardiovascular Disease Risk Factors Among Chinese Americans in New York City, 2009–2012

**DOI:** 10.5888/pcd14.160582

**Published:** 2017-05-11

**Authors:** Simona C. Kwon, Laura C. Wyatt, Shijian Li, Nadia S. Islam, Stella S. Yi, Chau Trinh-Shevrin

**Affiliations:** 1 NYU School of Medicine, New York, New York; 2 State University of New York, College at Old Westbury, Old Westbury, New York

## Abstract

We used the Racial and Ethnic Approaches to Community Health Across the US (REACH US) Risk Factor Survey from 2009 through 2012 to examine the association between body mass index (BMI, calculated as kg/m^2^) and 3 cardiovascular disease risk factors among Chinese Americans in New York City. We used traditional BMI cut points and cut points modified for the Asian population. Compared with normal/underweight Chinese American adults (BMI <23.0), obese Chinese American adults (BMI ≥27.5) had significantly higher odds of having each risk factor in fully adjusted logistic regression models: diabetes (odds ratio [OR], 4.2; 95% confidence interval [CI], 2.8–6.2), high blood pressure (OR, 5.5; 95% CI, 3.9–7.7), and high cholesterol (OR, 1.7; 95% CI, 1.2–2.4). Regression results were similar across BMI definitions, suggesting that both BMI categorizations should be considered in CVD research among Chinese Americans.

## Objective

Asian Americans are the fastest growing racial/ethnic group in the United States, and Chinese Americans are the largest subgroup (1). The prevalence of overweight and obesity is low among the Asian American population, compared with non-Hispanic white and black populations, when these terms are defined by standard body mass index (BMI) cut points (2). A World Health Organization panel proposed lowering BMI cut points for the Asian population because the association between BMI and health risks for the Asian population is different from the association for European populations (3). Chinese Americans are disproportionately affected by modifiable cardiovascular disease (CVD) risk factors such as diabetes and high blood pressure (4,5). Few studies have used BMI cut points modified for Asians in examinations of modifiable CVD risk factors among Asian Americans, including Chinese Americans (4). The objective of our study was to describe the association between BMI, using both standard and Asian-modified cut points, and modifiable CVD risk factors among Chinese Americans in New York City.

## Methods

Although racial/ethnic minority populations are increasing in the United States, large-scale community-based surveys for these populations are limited. The Racial and Ethnic Approaches to Community Health Across the US (REACH US) Risk Factor Survey was conducted annually from 2009 through 2012 in 28 grantee communities to evaluate projects in racial/ethnic minority communities. An address-based sampling method targeted areas and populations consistent with NYU School of Medicine’s B Free CEED grantee community (largely Chinese Americans living in New York City). In-person interviews, telephone interviews, and self-administered questionnaires were conducted. Chinese American respondents completed the survey in English, Mandarin, or Cantonese. Details on survey methods are available elsewhere (6).

Survey items were adapted from the Behavioral Risk Factor Surveillance System interview (7). Self-reported height and weight were used to calculate BMI as weight in kilograms divided by height in meters squared. We used standard BMI cut points (normal/underweight [<25.0]), overweight [25.0–29.99], and obese [≥30.0]) and cut points modified for the Asian population (normal/underweight [<23.0], overweight [23.0–27.49], and obese [≥27.5]) (3). Outcomes were self-reported diagnoses of diabetes, high blood pressure, and high cholesterol. Gestational diabetes was not considered a diabetes outcome, and diabetes type was not determined. Additional adjustment variables included sociodemographic and health-related characteristics: sex, age group, education, annual household income, nativity, health insurance (yes or no), most recent routine check-up (≤1 y ago or >1 y ago or never), self-reported CVD diagnosis, and smoking status.

In New York City, 3,001 adult (aged ≥18 y) survey respondents self-identified as Chinese American; 2,904 self-reported height and weight and were included in analyses. We conducted all analyses using SAS-callable SUDAAN software version 11.0 (RTI International) to account for the complex sample design (6). We tabulated descriptive statistics and generated prevalence estimates; outcomes were age-adjusted and calculated overall and by BMI category. *P* values were calculated by using PROC CROSSTAB. PROC LOGISTIC generated odds ratios (ORs) and 95% confidence intervals (CIs) for the association between BMI and each risk factor, adjusting for sociodemographic and health-related characteristics; significance was set at *P* < .05.

## Results

Eighty-six percent of the sample population was foreign-born, and one-third reported having less than a high school diploma (Table 1). The age-adjusted prevalence of overweight and obesity was 24.2% and 4.9%, respectively, using standard BMI cut points and 37.9% and 11.9%, respectively, using Asian-modified BMI cut points.

**Table 1:** Sociodemographic and Health-Related Characteristics of Chinese Americans (N = 2,904) Surveyed in the Racial and Ethnic Approaches to Community Health Across the US (REACH US) Risk Factor Survey, New York City, 2009–2012^a^

Characteristic	Value^b^	Unweighted No.^c^
**Sex **		
Male	49.4 (47.2–51.5)	1,244
Female	50.6 (48.5–52.8)	1,660
**Age, mean, y**	47.0 (46.4–47.7)	2,877
**Age group, y **		
18–24	12.1 (10.8–13.6)	241
25–44	34.7 (32.9–36.5)	815
45–64	35.2 (33.5–37.0)	1,036
≥65	18.0 (16.9–19.1)	785
**Nativity **		
US born	14.5 (13.1–16.0)	343
Foreign born	85.5 (84.0–86.9)	2,550
**Education **		
<High school diploma	32.2 (30.5–33.9)	1,049
High school diploma/Some college	34.8 (33.0–36.7)	953
College graduate	33.0 (31.2–34.7)	868
**Annual household income, $ **		
<25,000	55.1 (53.4–56.9)	1,566
25,000–49,999	21.3 (19.8–22.9)	531
≥50,000	23.6 (22.1–25.1)	572
**Health insurance **		
Yes	84.5 (83.0–85.8)	2,490
No	15.5 (14.2–17.0)	392
**Most recent routine check-up **		
≤1 y	72.9 (71.2–74.7)	2,214
>1 y or never	27.1 (25.4–28.8)	679
**Cigarette smoking **		
Current smoker	10.5 (9.3–11.8)	262
Former smoker	11.7 (10.6–13.1)	355
Never smoked	77.8 (76.1–79.4)	2,242
**Diagnosis of cardiovascular disease^d^**		
Yes	5.9 (5.1–6.8)	209
No	94.1 (93.2–94.9)	2,656

a Survey items were adapted from the Behavioral Risk Factor Surveillance System interview (7).

b All values are percentage (95% confidence interval) unless otherwise indicated. Percentages were weighted to reflect the probability of selection, the number of eligible family members, and the age–sex population size.

c Not all categories add to 2,904 because some respondents did not answer all survey questions.

d Ever received a diagnosis of a heart attack, angina/coronary heart disease, or a stroke.

The overall age-adjusted prevalence of diabetes was 9.2%; of high blood pressure, 23.2%; and of high cholesterol, 30.2% (Table 2). The prevalence of each risk factor increased incrementally across both sets of BMI categories, and differences in the prevalence of each risk factor were significant across both sets of BMI categories (Table 2).

**Table 2:** Relationship Between BMI and CVD Risk Factors Using Asian-Modified and Standard BMI Cut Points for Chinese American (N = 2,904) Survey Respondents Reporting BMI in The Racial and Ethnic Approaches to Community Health Across the US (REACH US) Risk Factor Survey, New York City, 2009–2012^a^

Variable	Total (N = 2,904)	BMI Cut Points Modified for Asian Population			Standard BMI Cut Points		
		BMI <23.0 (n = 1,436)	BMI 23.0–27.49 (n = 1,123)	BMI ≥27.5 (n = 345)	BMI <25.0 (n = 2,053)	BMI 25.0–29.99 (n = 706)	BMI ≥30.0 (n = 145)
**Self-reported diagnosis of diabetes**							
% (95% CI)	9.2 (8.3–10.1)	5.7 (4.7–6.8)^b^	10.2 (8.7–11.9)^b^	18.6 (15.3–22.5)^b^	6.8 (5.9–7.9)^b^	12.3 (10.3–14.7)^b^	23.1 (17.8–29.5)^b^
Age-adjusted, OR (95% CI)	—	1.0 [Ref]	2.0 (1.5–2.7)^b^	4.6 (3.3–6.6)^b^	1.0 [Ref]	2.1 (1.6–2.8)^b^	5.3 (3.5–8.2)^b^
Fully adjusted^c^, OR (95% CI)	—	1.0 [Ref]	1.8 (1.3–2.5)^b^	4.2 (2.8–6.2)^b^	1.0 [Ref]	1.9 (1.4–2.6)^b^	5.1 (3.2–8.2)^b^
**Self-reported diagnosis of high blood pressure**							
% (95% CI)	23.2 (21.9–24.7)	16.1 (14.5–17.9)^b^	25.6 (23.4–28.0)^b^	42.7 (37.6–48.1)^b^	18.6 (17.2–20.1)	29.8 (26.8–33.0)^b^	53.7 (45.4–61.8)^b^
Age-adjusted, OR (95% CI)	—	1.0 [Ref]	2.0 (1.7–2.5)^b^	5.6 (4.0–7.7)^b^	1.0 [Ref]	2.2 (1.8–2.7)^b^	8.0 (4.9–13.1)^b^
Fully-adjusted^c^, OR (95% CI)	—	1.0 [Ref]	1.9 (1.5–2.5)^b^	5.5 (3.9–7.7)^b^	1.0 [Ref]	2.1 (1.7–2.7)^b^	7.9 (4.8–13.1)^b^
**Self-reported diagnosis of high cholesterol**							
% (95% CI)	30.2 (28.6–31.9)	26.0 (23.8–28.2)^b^	32.3 (29.5–35.3)^b^	42.7 (36.5–49.1)^b^	27.7 (25.9–29.7)	35.8 (32.0–39.9)	41.9 (33.1–51.3)
Age-adjusted, OR (95% CI)	—	1.0 [Ref]	1.4 (1.2–2.7)^b^	2.0 (1.5–2.7)^b^	1.0 [Ref]	1.5 (1.2–1.8)^b^	1.7 (1.2–2.6)^d^
Fully adjusted^c^, OR (95% CI)	—	1.0 [Ref]	1.3 (1.0–1.6)^e^	1.7 (1.2–2.4)^d^	1.0 [Ref]	1.4 (1.1–1.7)^e^	1.5 (0.9–2.3)

Abbreviations: —, not applicable; BMI, body mass index; CI, confidence interval; CVD, cardiovascular disease; OR, odds ratio; Ref, reference.

a Survey items were adapted from the Behavioral Risk Factor Surveillance System interview (7). Percentages and odds ratios were weighted to reflect the probability of selection, the number of eligible family members, and the age–sex population size. BMI calculated as weight in kilograms divided by height in meters squared.

b *P* < .001.

c Fully adjusted model was adjusted for sex, age group, education, annual household income, health insurance (yes or no), most recent routine check-up (≤1 y ago or >1 y ago), self-reported CVD diagnosis, smoking, and nativity.

d *P* < .01.

e *P* < .05.

In fully adjusted logistic regression models, overweight and obesity determined by both sets of cut points were significantly associated with diabetes, high blood pressure, and high cholesterol (Table 2). Compared with normal/underweight adults, obese adults had significantly higher odds of having each risk factor: diabetes (odds ratio [OR], 4.2; 95% confidence interval [CI], 2.8–6.2], high blood pressure (OR, 5.5; 95% CI, 3.9–7.7], and high cholesterol (OR, 1.7; 95% CI, 1.2–2.4). We found similar results when we used standard BMI cut points; compared with normal/underweight adults, obese adults had significantly higher odds of diabetes (OR, 5.1; 95% CI, 3.2–8.2) and high blood pressure (OR, 7.9; 95% CI, 4.8–13.1), although high cholesterol (OR, 1.5; 95% CI, 0.9–2.3) was no longer significant.

## Discussion

Our findings expand on obesity and CVD research among Chinese Americans. The prevalence of overweight and obesity measured by using standard and Asian-modified cut points in our Chinese American sample was lower than the prevalence estimated in previous research (2,5,8,9). The prevalence of diabetes and high blood pressure in our sample, however, was higher than the prevalence determined in previous research (2,5,8). CVD risk factors are prevalent among Asian Americans at lower BMI levels than standard BMI levels (3,5). Our research showed that obese Chinese Americans (BMI ≥27.5) were significantly more likely to self-report a diagnosis of diabetes, high blood pressure, or high cholesterol than were Chinese Americans who were underweight or of normal weight (BMI <23.0). In general, the odds of self-reporting a diagnosis of a CVD risk factor were higher when we used standard BMI cut points than when we used Asian-modified cut points, but the association between BMI and each risk factor was significant across all Asian-modified cut points; obesity was not significantly associated with high cholesterol in the fully-adjusted model that used standard BMI cut points. Regression results were similar across BMI definitions, suggesting that both categorizations should be considered when conducting CVD research among Chinese Americans.

Our study has limitations. The self-reported data may result in underestimating or overestimating modifiable CVD risk factors and BMI (10,11). Data were cross-sectional, and causation cannot be inferred. Because of the small number of respondents who had a BMI of 30.0 or greater, 95% CIs were larger for this group than for the group with a BMI of 27.5 or greater. Finally, findings may only apply to Chinese immigrants in Asian enclaves in New York City. Surveys, however, were conducted in Chinese, enhancing the generalizability of these findings to English-speaking and non-English–speaking Chinese Americans in New York City.

Overweight and obesity are a priority health issue. Asian Americans have a higher risk of illness and death caused by chronic disease at lower BMI levels than non-Hispanic whites (4,8); thus prevalence estimates of disease based on standard categories of BMI underestimate the health burden associated with adiposity in Asian American adults (12). Using modified cut points may help to identify Asian Americans at risk for CVD and improve programs designed to prevent CVD among Asian American subgroups.
